# Evidence for Quark Confinement in the Proton

**DOI:** 10.34133/research.1369

**Published:** 2026-08-03

**Authors:** Xiangdong Ji, Gerald A. Miller, Chen Yang

**Affiliations:** ^1^ Tsung-Dao Lee Institute and School of Physics and Astronomy, Shanghai Jiao Tong University, Shanghai 201210, China.; ^2^ Department of Physics, University of Washington, Seattle, WA 98195-1560, USA.; ^3^ Maryland Center for Fundamental Physics, Department of Physics, University of Maryland, College Park, MD 20742, USA.

## Abstract

The strong interaction is the fundamental force that holds quarks and the gluon force carriers together to form protons and neutrons and also binds the atomic nucleus. The theory governing quark–gluon interactions is quantum chromodynamics (QCD). A wide variety of experimental data teaches us that quarks and gluons cannot be observed in isolation, a phenomenon known as confinement that is unique to QCD. But no one has used QCD to mathematically prove confinement. Here, we show how to define and measure the force on quarks in the proton using available experimental data. Direct evidence for confinement is obtained because the force is found to be attractive and constant for a wide range of quark positions. This work guides future experimental efforts at future electron-ion colliders aimed at obtaining a rigorous quantitative understanding of confinement and the origin of nuclear mass.

## Introduction

Rapid progress in understanding the role of the quark and gluon energy-momentum tensor (EMT) and the associated momentum conservation laws [1–4], along with the newly found ability to measure its properties [5–8], has recently occurred. But the link between these properties and the underlying mystery of quark confinement has not been established.

There are several important reasons for this. The difficulties can be understood by comparing the present situation with the history of the most prominent forces in our daily lives—gravity and electromagnetism. The inverse square laws of gravity and electricity were both discovered in table-top laboratory experiments by Cavendish and Coulomb using a torsion balance [9,10], but it is impossible to place quarks on a torsion balance and directly read the forces between them. This is because of the effects of confinement [11–13] that prevent the observation of a free quark. Furthermore, in the small-sized world of the proton, the uncertainty principle precludes finding quarks at a definite position. To make matters even more difficult, the quarks within the proton move at speeds very close to those of light. Einstein’s theory of relativity must be respected.

Thus, the fundamental strong forces acting between colored quarks have never been accessed by direct experimental measurement, but instead are postulated within a theory known as quantum chromodynamics (QCD) that operates in the sub-atomic quantum domain. The interactions between quarks separated by small distances, namely, asymptotic freedom [14,15], have been studied using high-energy scattering, but at larger distances, our knowledge is largely limited to models of hadron spectroscopy [16–18] and models of static potentials for massive quarks [19]. Finding a way to measure the forces on quarks that is directly connected to experimental measurements is therefore necessary to study inter-quark forces at large separations without using models. If this is accomplished, a test of the fundamental theory and understanding of the QCD confinement mechanism would be obtained.

In the following, we show how to overcome the various roadblocks mentioned above. The first step is to understand the proper definition of forces. This is done using the QCD EMT, the strong-interaction version of the well-known Maxwell stress tensor used to understand electromagnetic forces. The nucleon matrix element of the EMT is defined in terms of kinematic factors and relativistically invariant form factors with the specific forms chosen so that the conservation of energy and momentum is respected [1]. Then, one needs to obtain the form factors from various experimental measurements [20–22] and fundamental numerical calculations known as lattice QCD [11]. This information allows a force density to be obtained. The force itself is obtained by dividing its density by the density of quarks [23], with the latter also obtained from experimental measurements. All this is not yet enough. It is necessary to obtain an unambiguous way to define the position of a quark that respects the theory of relativity and the uncertainty principle. This is done by making evaluations in an infinite momentum frame (IMF) [24–26]. The net result is a well-defined expression for the force on quarks in a proton that can be evaluated using the relevant input information available.

## Results

### Force density in the proton

We shall extend a method to extract the force on quarks that has been well-tested in nonrelativistic, quantum mechanical systems [27]. Force densities are obtained from the divergence of the EMT [28], which receives contributions from quarks, gluons, and an anomalous term [2]. For a stable system, the divergence of the total EMT vanishes, but the individual terms have nonvanishing divergences, giving rise to force components. Our interest here is in the forces on the quarks. The quark (*q*) part of the EMT is traceless and is given by

$$T_{q}^{\mu \nu }=\frac{1}{2}\bar{\psi }i\overset{↔}{D}\;{\;}^{(\mu }\gamma^{\nu )}\psi ,$$ (1)

The divergence of this EMT represents the local momentum change of the quarks. Analogous to Newton’s law, it thus yields [3,4] the density of the forces acting on the quarks:

$$F_{q}^{i}\equiv \partial_{\mu }T_{q}^{\mu i}=g\bar{\psi }\gamma_{\mu }F^{\mu i}\psi$$ (2)

$$=g\bar{\psi }\gamma_{0}F^{0i}\psi +g\bar{\psi }\gamma_{j}F^{\mathrm{ji}}\psi$$ (3)

$$=g{\left(\rho_{c}{\vec{E}}_{c}+{\vec{j}}_{c}\times {\vec{B}}_{c}\right)}^{i}$$ (4)

where *c* is the color index, *i* represents a spatial variable, *g* is the strong coupling constant, $\left\{\rho_{c},\;{\vec{j}}_{c}\right\}$ are the quark color-charge and color-current densities, and $\left\{{\vec{E}}_{c},\;{\vec{B}}_{c}\right\}$ are the chromo-electromagnetic fields. The right-hand side of Eq. (4) is the gluon version of the Lorentz force law, or the color-Lorentz force [3,4], familiar from electromagnetism.

Nucleon momentum densities and flows are measured through high-energy experiments as the matrix elements of the EMT between eigenstates of momentum, parametrized in terms of kinematic terms and EMT form factors, expressed as $\left\{A,B,C,\bar{C}\right\}$ [1]. These are experimentally accessible through exclusive processes such as deeply virtual Compton scattering (DVCS) and deep exclusive meson production (DVMP) [1,5–8,20–22,29–33]. The form factors that describe the EMT can also be computed using first-principles lattice-QCD (LQCD) calculations [34].

The force density, $F_{q}^{j}$, as shown in Eq. (4), is obtained from the term in the quark part of the EMT with a nonvanishing divergence; these are denoted as nonconserved (nc). The conserved terms have vanishing divergence and thus give no contribution to the force density. The relevant term is the ${\bar{C}}_{q}$ form factor in the matrix elements [1],

$$\begin{array}{c} {\langle P^{\prime }|T_{q}^{\mu \nu }|P\rangle }_{\mathrm{nc}}=\bar{U}\left(P^{\prime }\right)\left[g^{\mu \nu }M{\bar{C}}_{q}\left(q^{2}\right)\right]U\left(P\right)\\ \;=\bar{U}\left(P^{\prime }\right)\left[-\frac{1}{4}g^{\mu \nu }{\mathrm{MG}}_{s,\;q}\left(q^{2}\right)\right]U\left(P\right), \end{array}$$ (5)

where *U* and $\bar{U}$ are proton momentum-spin wave functions, *M* is the proton mass, $q^{\mu }={P^{\prime }}^{\mu }-P^{\mu }$ is the momentum transfer, and ${\bar{C}}_{q}\left(q^{2}\right)=-G_{s,\;q}\left(q^{2}\right)/4$ is the quark scalar form factor obtained from the constraint that the quark EMT is traceless [35],

$$G_{s,\;q}\left(q^{2}\right)=A_{q}\left(q^{2}\right)+B_{q}\left(q^{2}\right)\frac{q^{2}}{4M^{2}}-C_{q}\left(q^{2}\right)\frac{3q^{2}}{M^{2}}\;,$$ (6)

with $q^{2}={\left(P^{\prime }-P\right)}^{2}$. The form factor $B_{q}\left(q^{2}\right)$ is relatively small and is therefore neglected in the following calculations. See the Supplementary Materials for details on the matrix elements and EMT form factors.

### Relativistic position variable respecting uncertainty principle

Experimental data and LQCD only measure the matrix elements of the EMT and force density $F_{q}^{j}$ between momentum states, as shown in Eq. (5), but such states do not have a well-defined position. Furthermore, the different momenta of the initial and final states lead to different internal dynamics and structures due to relativity, while a true density is defined by a matrix element between the same states.

**Fig. 1.**
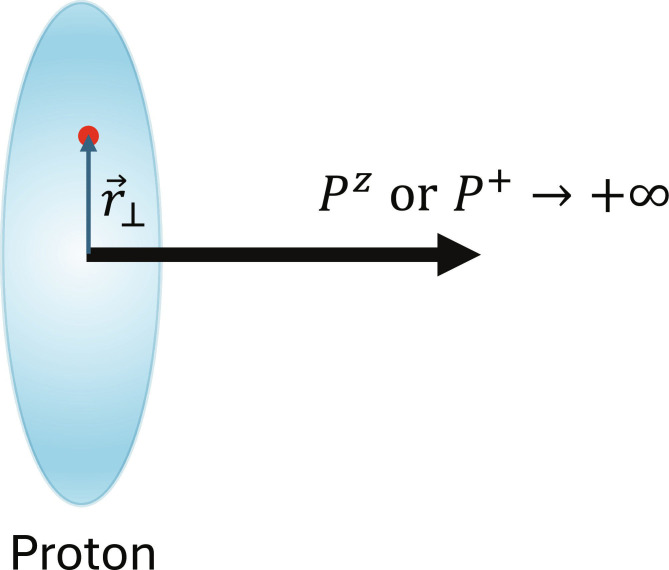
Proton traveling in the infinite-momentum frame. The proton is localized in the transverse plane with a well-defined transverse center as the origin. The transverse positions ${\vec{r}}_{⊥}$ are defined relative to the origin with associated spatial distributions in this transverse plane.

To handle this, the proton state vector, for both the initial and final states, is taken to be an infinite superposition of states with fixed infinite momentum in one direction (*z*) and any momentum transverse to *z*: We use an infinite momentum frame [24–26]. In this frame, the transverse momentum of the system factorizes out of the wave function so that states of any momentum transverse to *z* have the same internal dynamics. This superposition yields a state of well-defined transverse position that can be taken to be the origin so that transverse positions ${\vec{r}}_{⊥}$ relative to the origin are defined with the associated spatial distributions in this transverse plane [36–38], as shown in Fig. 1. The nonconserved part of the quark EMT in the transverse plane is then

$${\langle T_{q}^{\mu \nu }\rangle }_{\mathrm{nc}}\left({\vec{r}}_{⊥}\right)=-\frac{1}{4}g^{\mu \nu }{\mathrm{MG}}_{s,\;q}\left({\vec{r}}_{⊥}\right)$$ (7)

which is given by the scalar form factor, and its spatial density $G_{s,\;q}\left({\vec{r}}_{⊥}\right)$ is obtained by Fourier–Bessel transforming $G_{s,\;q}\left(-q_{⊥}^{2}\right)$. Details are provided in the Supplementary Materials.

In the 4-dimensional divergence appearing in Eq. (4), 2 of these derivatives $\partial_{+}$ and $\partial_{-}$ vanish when matrix elements are taken between the proton state vector. This is because the energy transfer vanishes because $P^{+}\to \infty$, and the longitudinal momentum is fixed at an infinite value. On the other hand, the nonconserved part is proportional to $g^{\mu \nu }$ as in Eq. (7). Therefore, since $g^{+i}=g^{-i}=0$, only the transverse derivative survives, as shown in Eq. (S20). Thus, we are left with only the transverse parts of the divergence. This means that the force density is simply

$$F_{q}^{i}\left({\vec{r}}_{⊥}\right)=\partial_{\mu }\langle T_{q}^{\mu i}\rangle \left({\vec{r}}_{⊥}\right)=\frac{M}{4}\nabla_{⊥}^{i}G_{s,\;q}\left({\vec{r}}_{⊥}\right)$$ (8)

See Eqs. (S23) and (S24) for an explicit mathematical derivation.

### Measuring the strong forces acting on quarks in the proton

The force on the quarks is the force density, Eq. (8), divided by the quark density, $\rho_{q}\left(r_{⊥}\right)$. The function $\rho_{q}\left(r_{⊥}\right)$ can be obtained, using the infinite momentum frame discussed above, by taking the 2-dimensional Fourier transform of the sum of the proton and neutron Dirac form factors $3\left[F_{1}^{p}\left(q^{2}\right)+F_{1}^{n}\left(q^{2}\right)\right]$ [37], where the factor 3 reflects the number of valence quarks. Therefore, the color-Lorentz force on quarks is given by

$${\vec{F}}_{q}\left({\vec{r}}_{⊥}\right)\equiv \frac{{\vec{F}}_{q}\left({\vec{r}}_{⊥}\right)}{\rho_{q}\left({\vec{r}}_{⊥}\right)}=\frac{\left(M/4\right){\vec{\nabla }}_{⊥}G_{s,q}\left({\vec{r}}_{⊥}\right)}{3\left[F_{1}^{p}\left({\vec{r}}_{⊥}\right)+F_{1}^{n}\left({\vec{r}}_{⊥}\right)\right]}\;,$$ (9)

where the spatial densities are obtained by Fourier–Bessel transformations of the corresponding form factors. See the Supplementary Materials for details. Due to rotational symmetry in the transverse plane, the force lies exclusively along the radial direction.

Equation (9) shows how to precisely determine the force once all the form factors are known for a large range of values of the momentum transfer. The accuracy of our force determination depends on the current incomplete nature of this knowledge.

**Fig. 2.**
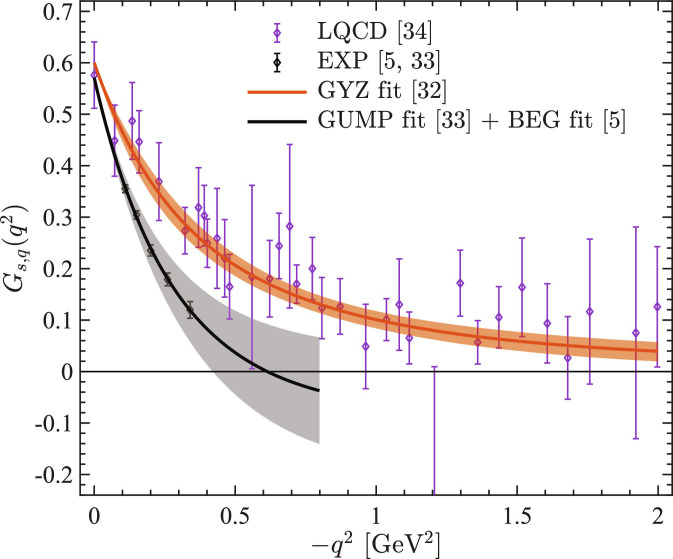
The quark scalar form factor $G_{s,q}\left(q^{2}\right)$ obtained from LQCD calculations and experimental data. Direct LQCD calculations are shown as purple error bars [34]. The GYZ fit (orange curve and band) represents the Bayesian inference of LQCD (main) and J/ψ photoproduction data [32]. The black error bars, curve, and band combine the GUMP fit and BEG’s dispersive analysis of DVCS data, which mainly exhibit the experimental information and receive minor constraints from LQCD [5,33].

We perform a comprehensive assessment of the state-of-the-art global phenomenological analyses, including both LQCD and experimental inputs to extract the quark scalar form factor [5,6,32–34]; the results are summarized in Fig. 2. The LQCD calculations of the EMT form factors (using pion mass $m_{\pi }=169$ MeV, and lattice spacing *a* = 0.091 fm) are shown as data points with purple error bars [34]. The $A_{q}\left(q^{2}\right)$-form factor, which appears as part of $G_{s,\;q}\left(q^{2}\right)$, is rescaled so that $A_{q}\left(0\right)$ of LQCD is consistent with the Coordinated Theoretical-Experimental Project on QCD (CTEQ) extraction [39]. The GYZ fit (orange curve with its uncertainty band) is a Bayesian analysis driven primarily by lattice constraints, with only weak sensitivity to near-threshold J/ψ photo-production measurements [32], as shown in figure 3 of Ref. [32]. The experimental information is mainly reflected by the black curve and band, obtained by combining the global extraction of generalized parton distributions (GUMP fit) [33] and the dispersive analysis of DVCS data (BEG fit) [5]. Due to the limited kinematic range available for current experiments, the inferred scalar form factor is poorly constrained at large values of momentum transfer, $|q^{2}|$. See the Supplementary Materials for numerical details and error analyses. Note that the current GUMP fit does not incorporate an error estimate for $A_{q}\left(q^{2}\right)$, and the uncertainties of $G_{s,\;q}\left(q^{2}\right)$ and the force below solely come from the BEG fit of $C_{q}\left(q^{2}\right)$.

**Fig. 3.**
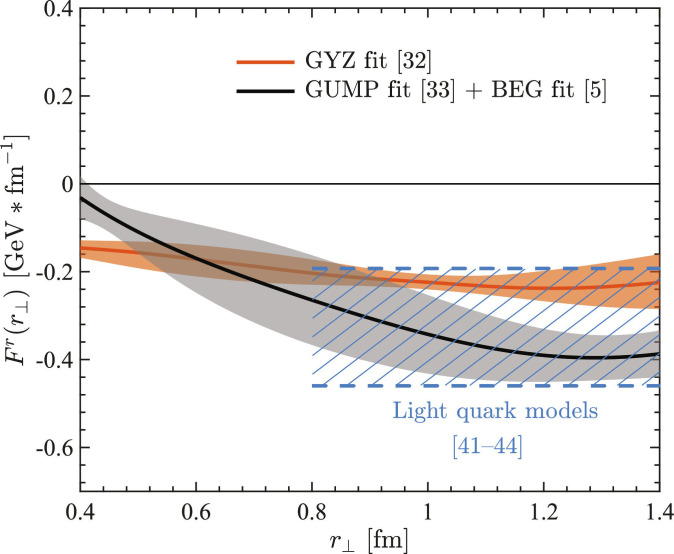
Strong force on quarks in the proton in the transverse plane of the IMF. The forces are obtained using the latest global analyses of the quark momentum current form factors [5,32,33]. The figure shows the total force from experimental data (black curve and band) and from the fit dominated by LQCD calculations (orange curve and band). The blue hatched band shows the string tensions from several relativistic quark models, which spans from 0.19 GeV/fm to 0.46 GeV/fm [41–44].

The force acting on the proton’s quarks in the IMF, using Eq. (9), is displayed in Fig. 3. The denominator of Eq. (9) is obtained from the Dirac proton and neutron form factors of Ref. [40]. The forces extracted from the GYZ fit and GUMP and BEG fits, together with the 1σ deviations, are shown as the orange and black curves and associated shaded areas. For comparison, we also show the constant confining force inferred from several relativistic quark models as the blue hatched band [41–44].

The experimental results from GUMP and BEG fits provide strong evidence for a net confining force acting on quarks. At intermediate distances, specifically $r_{⊥}=1.0∼1.4$ fm, the total force acting on the quarks extracted from the experimental data is consistent with the QCD linear potential within uncertainties. Averaging over this interval yields ${\bar{F}}_{q}=-0.382\left(92\right)$ GeV/fm, where the uncertainty corresponds to a 1σ deviation. With more data availability in the future, a rigorous statistical analysis of this constant force behavior can be studied. On the other hand, the GYZ fit, dominated by the LQCD data, produces a mean value ${\bar{F}}_{q}=-0.217_{-0.015}^{+0.016}$ GeV/fm with uncertainties 1σ, for the range $r_{⊥}=0.7∼1.2$ fm. The GYZ fit exhibits relatively small uncertainties, obtained from a Monte Carlo replica method. Note that these uncertainties may be underestimated as the systematic errors in the lattice calculations are not thoroughly considered. These values are also compatible with relativistic quark models [41–44]. The negative value of the force denotes an attractive potential on the quark because the force is the negative gradient of the potential.

Extending the curves to larger values of $r_{⊥}$ than shown in Fig. 3 would involve a substantial increase in the uncertainties, particularly for experimental extraction. This illustrates the challenges in measuring the EMT form factors. These can only be accessed through processes sensitive to generalized parton distributions [1,21]. Measuring these processes generally requires data from high-energy and high-luminosity facilities with detectors capable of precise and complete mapping of scattering events. Therefore, data from the future electron-ion colliders [45,46] are crucially needed to improve the precision of EMT form factors and thereby achieve a rigorous quantitative understanding of color confinement.

## Discussion and Conclusion

The approximately constant strong force on the component quarks observed far away from the proton center that we find is consistent with the known QCD linear potential acting between heavy quarks. This confinement force is about half the QCD string tension σ≈0.9GeV/fm modeled from heavy quarkonium. This reduction is expected because the motion of the light quarks that make up the proton spreads out the postulated flux tube between the quarks [19] as is also indicated by the hopping expansion [47]. A lower string tension for light quarks is also consistent with the findings in light mesons [48].

We note that the GYZ fit, based mainly on LQCD results, yields a weaker confining force than the one inferred from the experimental data. This difference is plausibly attributable to various lattice artifacts such as excited state contamination, the large pion mass, and the finite lattice spacings used, as $A_{q}\left(0\right)$ shows a significant deviation from the data.

In summary, we show how to directly determine the strong force on quarks within hadrons using momentum current distributions and associated form factors in Eq. (9) accessible through high-energy experiments and lattice QCD calculations. By applying our approach to the proton in the infinite-momentum frame, we obtain the strong-interaction force acting on quarks and strong evidence for quark confinement. The total confinement force is consistent with the linear potential nature of QCD, although with significant experimental uncertainties. The future electron-ion colliders and other experiments will significantly improve this extraction by extending the kinematic coverage of the DVCS process up to *Q*^2^ ∼ 10^3^ GeV^2^ and expanding the momentum transfer $|q^{2}|$ down to 0.02 GeV^2^ and Bjorken-*x* range down to 10^−4^. This will significantly reduce the uncertainties in the extracted quark scalar form factor $G_{s,\;q}$ in the large-$r_{⊥}$ region, enabling a high-precision tomography of the quark confinement force.

## Materials and Methods

Details of the materials and methods used in this article are available in the Supplementary Materials.

## Data Availability

The data used to support the findings of this study are available from the authors upon request.
