# Supplementary material for: Evidence for Quark Confinement in the Proton
Source: Research (Wash D C). 2026 Aug 3;9:1369. doi: 10.34133/research.1369 (PMC13429911; doi:10.34133/research.1369)
Supplement: Supplementary 1 — Materials and Methods [file research.1369.f1.pdf]

Supplementary Materials

Force Density Form Factors in the Proton

In relativistic quantum field theories, particularly the nucleon in QCD, extracting the quark confinement force involves the quark force density and probability density in hadrons. These physical quantities can be expressed in terms of several form factors in hadrons using the following method. The key expression is Eq. (9).

The numerator of Eq. (9), the quark force density, is obtained by taking the divergence of the QCD quark EMT, including momentum densities and flows. The divergence of the quark EMT at the operator level yields

$$\mathcal{F}_q^j \equiv \partial_\mu T_q^{\mu j} = g\bar{\psi}\gamma_\mu F^{\mu j}\psi = \sum_{c=1}^{8} g(\rho_c \vec{E}_c + \vec{j}_c \times \vec{B}_c)^j , \qquad (10)$$

where we have used the QCD equation of motion. The final expression is the color-Lorentz force density acting on quarks.

The nucleon momentum densities and flows are measured through high-energy experiments as the matrix elements of the QCD EMT in the momentum states, parametrized in terms of several EMT form factors,

$$\langle P' | T_q^{\mu\nu} | P \rangle = \bar{U}(P')\left[A_q(q^2)\gamma^{(\mu}\bar{P}^{\nu)} + B_q(q^2)\frac{\bar{P}^{(\mu}i\sigma^{\nu)\alpha}q_\alpha}{2M} + C_q(q^2)\frac{q^\mu q^\nu - q^2 g^{\mu\nu}}{M} + M\bar{C}_q(q^2)g^{\mu\nu} \right]U(P) , \qquad (11)$$

The above definition of the force density indicates that it is only related to the non-conserved (nc) part (the part with non-zero divergence) of the quark EMT and the associated form factor given by $\bar{C}_q(q^2)$ [1]. Furthermore, since the quark EMT is traceless, one finds $\bar{C}_q(q^2) = -G_{s,q}(q^2)/4$ and,

$$\langle P' | T_q^{\mu\nu} | P \rangle_{nc} = \bar{U}(P')\left[-\frac{1}{4} g^{\mu\nu} M G_{s,q}(q^2) \right]U(P) , \qquad (12)$$

where M is the proton mass, $q^\mu = P'^\mu - P^\mu$ is the momentum transfer, and $G_{s,q}(q^2)$ is the quark scalar form factor,

$$G_{s,q}(q^2) = A_q(q^2) + B_q(q^2)\frac{q^2}{4M^2} - C_q(q^2)\frac{3q^2}{M^2} . \qquad (13)$$

Thus, obtaining the quark force density only requires this quark scalar form factor. The $B_q(q^2)$-form factor is relatively small and is thus neglected.

The denominator of Eq. (9), the quark probability density is approximated by using the Dirac proton and Dirac neutron form factors in the following way. In the IMF, proton and neutron's charge densities are related to the Dirac form factors [37], which can be approximated using the *u*- and *d*-quark densities in the proton,

$$\rho_p = F_1^p \approx \frac{2}{3}\rho_u - \frac{1}{3}\rho_d , \; \rho_n = F_1^n \approx \frac{2}{3}\rho_d - \frac{1}{3}\rho_u , \qquad (14)$$

where the second equation is obtained using isospin symmetry. Using these two equations, one can approximate the quark probability density in the proton as

$$\rho_q \approx \rho_d + \rho_u \approx 3(F_1^p + F_1^n) . \qquad (15)$$

The quark density is extracted by taking the two-dimensional Fourier transform of these form factors as discussed in the following section. In summary, one uses Eqs. (12) and (15) as inputs to the numerator and denominator of Eq. (9).

Spatial Densities in the Infinite-Momentum Frame

Since form factors are defined in the momentum space, exploring the related spatial distributions requires care. In quantum field theories, spatial distributions are defined as the expectation values of local operators $\mathcal{O}(x)$ in a localized single-hadron state $|\Psi_\sigma\rangle$. By constructing the localized state using a superposition of hadron momentum eigenstates with a Gaussian wave packet, one can derive the relation between the form factors and the spatial distributions.

The time dependence x^+ naturally vanishes in the infinite momentum frame defined by $P^+ \to \infty$, aligning with Feynman's motivation for creating the parton model. Then a Gaussian wave packet with spatial width σ in the transverse plane is used to define the localized state $|\Psi_\sigma\rangle$,

$$\langle p^+, \vec{p}_\perp | \Psi_\sigma \rangle = \sqrt{2\pi}(2\sigma) e^{-\sigma^2 \vec{p}_\perp^2} \sqrt{2p^+(2\pi)\delta(p^+ - P^+)}. \tag{16}$$

The inverse spatial width must be much smaller than the momentum of the hadron $1/\sigma \ll P^+$. The limit $\sigma \to 0$ is to be taken at the end of the calculation. The 2D spatial density is then obtained as the expectation value of the operator in this localized state, related to the form factors by [38],

$$\begin{aligned}\rho_{\mathcal{O}}(\vec{r}_\perp) &\equiv \lim_{P^+\to\infty} \int dr^- \langle \Psi_\sigma | \hat{\mathcal{O}}(r^+, r^-, \vec{r}_\perp) | \Psi_\sigma \rangle \\ &= (2\pi)(2\sigma)^2 \int \frac{d^2\vec{P}_\perp}{(2\pi)^2} e^{-2\sigma^2 \vec{P}_\perp^2} \int \frac{d^2\vec{q}_\perp}{(2\pi)^2} \frac{\langle P^+, \vec{p}_\perp' | \hat{\mathcal{O}}(0) | P^+, \vec{p}_\perp \rangle}{2P^+} e^{-\sigma^2 \vec{q}_\perp^2/2} e^{-i\vec{q}_\perp \cdot \vec{r}_\perp} ,\end{aligned} \tag{17}$$

where $\vec{P}_\perp$ is the average transverse momentum, $\vec{q}_\perp = \vec{p}_\perp' - \vec{p}_\perp$ is the momentum transfer in the transverse plane and the integral over r^- ensures that the initial and final states have the same P^+ . If the matrix elements only depend on the momentum transfer $\vec{q}_\perp$, one can integrate over the momenta and take the local limit $\sigma \to 0$ in the above formula, and now the Fourier transformation of the matrix elements acquires clear physical interpretations as the 2D spatial densities,

$$\lim_{\sigma\to 0} \rho_{\mathcal{O}}(\vec{r}_\perp) = \frac{M}{P^+} \int \frac{d^2\vec{q}_\perp}{(2\pi)^2} \frac{\langle P^+, \vec{p}_\perp' | \hat{\mathcal{O}}(0) | P^+, \vec{p}_\perp \rangle}{2M} e^{-i\vec{q}_\perp \cdot \vec{r}_\perp} , \tag{18}$$

This is the case for the proton and neutron's Dirac form factors $F_1^p(q^2)$ and $F_1^n(q^2)$. Using the above formula, the quark probability density on the transverse plane $3[F_1^p(\vec{r}_\perp) + F_1^n(\vec{r}_\perp)]$ is obtained by Fourier-Bessel transforming the Dirac form factors of the proton and neutron,

$$\rho_q(\vec{r}_\perp) = 3[F_1^p(\vec{r}_\perp) + F_1^n(\vec{r}_\perp)] = \int \frac{d^2\vec{q}_\perp}{(2\pi)^2} 3[F_1^p(-\vec{q}_\perp^2) + F_1^n(-\vec{q}_\perp^2)] e^{-i\vec{q}_\perp \cdot \vec{r}_\perp} \tag{19}$$

However, the EMT operators also contain terms in the matrix elements that depend on both $\vec{P}_\perp$ and $\vec{q}_\perp$, which, after integrating over the average transverse momentum, contribute as a ratio between the Compton wavelength $1/M$ and the localization scale σ. These terms are divergent in the localized wave packet limit $\sigma \to 0$, and therefore could render the interpretation as 2D

spatial densities ambiguous.

Fortunately, these physically ambiguous terms appear in the EMT operators as the conserved contributions, which, after taking the divergence to obtain the force density, precisely vanish. The non-conserved part is only related to the quark scalar form factor $G_{s,q}(q^2)$ contributing as a diagonal term proportional to $g^{\mu\nu}$. Thus, the 4-dimensional divergence reduces to the 2-dimensional divergence in the transverse plane and is independent of the average momentum $\vec{P}_\perp$. We omit the higher-twist factor M/P^+ in the transverse EMT, and the force density is thus obtained as,

$$\begin{aligned}\mathcal{F}_q^j(\vec{r}_\perp) &\equiv \partial_\mu \langle T_q^{\mu j}\rangle_{nc}(\vec{r}_\perp)\\ &= \nabla_\perp^i \langle T_q^{ij}\rangle_{nc}(\vec{r}_\perp) = \frac{M}{4}\nabla_\perp^j G_{s,q}(\vec{r}_\perp),\end{aligned} \tag{20}$$

where $i, j = x, y$ and the subscript nc represents the non-conserved part. The above spatial density is obtained similarly by taking the 2D Fourier-Bessel transformation of the quark scalar form factor in the momentum space,

$$G_{s,q}(\vec{r}_\perp) = \int \frac{d^2\vec{q}_\perp}{(2\pi)^2} G_{s,q}(-\vec{q}_\perp^2) e^{-i\vec{q}_\perp\cdot\vec{r}_\perp} \tag{21}$$

One may also explicitly examine this cancellation by calculating the divergence of the EMT,

$$\partial_\mu \langle T_q^{\mu j}\rangle = \partial_- \langle T_q^{-j}\rangle + \partial_+ \langle T_q^{+j}\rangle + \partial_i \langle T_q^{ij}\rangle\,. \tag{22}$$

Since the initial and final states have the same P^+, the x^- dependence vanishes and thus the first term does not contribute. The other two components can also be computed directly, by taking the limit $P^+ \to \infty$ in the final step,

$$\partial_+ \langle T_q^{+j}\rangle = \frac{1}{4\sigma^2 P^+}\left[-\nabla_\perp^j \int \frac{d^2\vec{q}_\perp}{(2\pi)^2} e^{-\sigma^2\vec{q}_\perp^2/2} e^{-i\vec{q}_\perp\cdot\vec{x}_\perp} A_q\left(-\vec{q}_\perp^2\right)\right] + \mathcal{O}\left(\frac{1}{\left(P^+\right)^3}\right), \tag{23}$$

$$\partial_i \langle T_q^{ij}\rangle = \frac{1}{4\sigma^2 P^+}\left[+\nabla_\perp^j \int \frac{d^2\vec{q}_\perp}{(2\pi)^2} e^{-\sigma^2\vec{q}_\perp^2/2} e^{-i\vec{q}_\perp\cdot\vec{x}_\perp} A_q\left(-\vec{q}_\perp^2\right)\right] + \mathcal{O}\left(\frac{1}{\left(P^+\right)^3}\right) + \partial_i \langle T_q^{ij}\rangle_{nc}\,.$$

(24)

The sum of the two divergent terms in the $\sigma \to 0$ limit cancel to order $(1/P^+)^3$, and one is left with the 2D derivative over the scalar form factor, which is free of ambiguities. Such cancellation is expected as it simply reflects that the divergence of the conserved parts in the EMT vanishes.

Numerical Demonstration

The numerical demonstration utilizes the parametrization of form factors, where the parameters are obtained from the latest global analysis including both experimental data and lattice QCD calculations.

The proton and neutron's Dirac form factors are related to the electric and magnetic form factors as,

$$F_1^{(p,n)}(q^2) = \frac{G_E^{(p,n)}(q^2) + \tau G_M^{(p,n)}(q^2)}{1+\tau}\,, \tag{25}$$

where $\tau = -q^2/4M^2$. These electric and magnetic form factors are parametrized using the formula,

$$\mathbb{G}(q^2) = \frac{1+\sum_{k=1}^{n} a_k \tau^k}{1+\sum_{k=1}^{n+2} b_k \tau^k}, \quad G_E^n(q^2) = \frac{A\tau}{1+B\tau}\frac{1}{(1-q^2/\Lambda^2)^2}, \tag{26}$$

where n=1, the form factors are $\mathbb{G} = \{G_E^p, G_M^p/\mu_p, G_M^n/\mu_n\}$, μ_p, μ_n are the magnetic moments of the proton and neutron, the parameter is $\Lambda^2 = 0.71 GeV^2$ and the other parameters $\{a_k, b_k, A, B\}$ can be found in Ref. [40]. Moreover, uncertainties in the Dirac form factors are small and thus neglected.

In the GYZ fits, the EMT FFs are parameterized using the multipole ansatz with three parameters $\{\alpha_{\mathbb{F}}, \mathbb{F}(0), M_{\mathbb{F}}\}$ as

$$\mathbb{F}(q^2) = \mathbb{F}(0)(1-q^2/M_{\mathbb{F}}^2)^{-\alpha_{\mathbb{F}}}, \tag{27}$$

where we choose $\alpha_{\mathbb{F}} = 2$ (dipole) for the $A_q(q^2)$ form factor and $\alpha_{\mathbb{F}} = 3$ (tripole) for the $C_q(q^2)$ form factor. The $B_q(q^2)$-form factor is relatively small and will be neglected. By performing the Fourier transformation with respect to the momentum transfer in the IMF $q^2 = -\vec{q}_\perp^{\,2}$, one obtains the spatial distributions $\mathbb{F}(\vec{r}_\perp)$. The scalar form factor in Eq. (6) then becomes

$$G_{s,q}(\vec{r}_\perp) = A_q(\vec{r}_\perp) - \frac{3}{M^2}\nabla_\perp^2 C_q(\vec{r}_\perp) \tag{28}$$

We use $N = 2\times10^4$ parameter sets obtained from the Monte-Carlo Markov Chain (MCMC) sampling method [32] to estimate the uncertainties in the extracted scalar form factor and the force,. We then propagate these MCMC samples utilizing the form factor and force formulas in terms of these parameters, through which the full parameter correlations are preserved. We take the median as orange curves shown in Fig. 2 and 3, and uncertainties are estimated using 1σ confidence intervals, shown as orange shaded areas.

On the other hand, a different parametrization is adopted for the EMT FFs extracted from the experimental data. The $A_q(q^2)$-form factor is obtained from the global extraction of generalized parton distributions, or the GUMP fit [33], while the $C_q(q^2)$-form factor is extracted from BEG's dispersive analysis of DVCS data [5].

In the GUMP fit, the conformal moments of generalized parton distributions are parametrized, where the first conformal moment of $H_q(x,\xi,q^2)$ reduces to the familiar Mellin moment, and thus EMT FFs [33],

$$\int_{-1}^{1} dx\, xH_q(x,\xi,q^2) = A_q(q^2) + 4\xi^2 C_q(q^2) \equiv \mathcal{F}_{1,0}^q(q^2) + \xi^2 \mathcal{F}_{1,2}^q(q^2), \tag{29}$$

where $\mathcal{F}_{1,0}^q(q^2), \mathcal{F}_{1,2}^q(q^2)$ are parametrized and correspond to $A_q(q^2), C_q(q^2)$ form factors, respectively. Note that since the ξ dependence in the above moment is neglected—$\mathcal{F}_{1,2}^q$ is not implemented in the parameterization—it only yields the A_q-form factor, which is parametrized as,

$$A_q^f(q^2) = \mathcal{F}_{1,0}^f(q^2) = \sum_{i=1}^{i_{\max}} N_{i,f} \frac{B(2-\alpha_{i,f}-\alpha'_{i,f}q^2, 1+\beta_{i,f})}{B(2-\alpha_{i,f}, 1+\beta_{i,f})} R_{i,f}(q^2) \quad (30)$$

where $f = u,d,\bar{u},\bar{d},g$ represents the flavor, $B(x,y)$ is the beta function, $\{N_{i,f}, \alpha_{i,f}, \beta_{i,f}, \alpha'_{i,f}\}$ are parameters obtained by the GUMP fit, $i_{\max} = 1$ for valence quarks *u,d* and $i_{\max} = 2$ for sea quarks and gluons $\bar{u},\bar{d},g$, $R_{i,f}(q^2) = (1-q^2/M_{i,f}^2)^{-2}$ for *u,d* and $R_{i,f}(q^2) = \exp(-b_{i,f}q^2)$ for $\bar{u},\bar{d},g$. The total $A_q(q^2)$-form factor is obtained by summing over all of the quark flavors.

The C_q-form factor is extracted by the BEG fit using a dispersive analysis of the DVCS data of beam-spin asymmetry and unpolarized cross sections. This form factor is also parametrized using a multipole ansatz with three parameters $\{\alpha_{\mathbb{F}}, \mathbb{F}(0), M_{\mathbb{F}}\}$ as in Eq. (27) being fitted in Ref. [5].

Combining the $A_q(q^2)$-form factor from the GUMP fit and $C_q(q^2)$-form factor from the BEG fit as well as the Dirac form factors, we plot the quark scalar form factor in Fig. 2 and the quark force in Fig. 3 as the black curves. Uncertainties are estimated by ignoring parameter correlations and adding errors of parameters in quadrature. Specifically, for a function $f(r;\{A_i\})$ depending on several parameters $\{A_i\}$ with corresponding errors $\{\Delta A_i\}$, the 1σ uncertainties are,

$$\Delta f(r;\{A_i\}) = \sqrt{\sum_i \left(\frac{\partial f(r;\{A_i\})}{\partial A_i} \Delta A_i \right)^2} \quad (31)$$

In the case of quark scalar form factor and force density, since the error analysis is not implemented in the current GUMP fit, uncertainties arise mainly from the $C_q(q^2)$-form factor extracted from BEG's fit, shown as the gray shaded area in Fig. 2 and 3. Note that it has been argued that BEG's fit has underestimated the overall uncertainties due to its model-dependence and several assumptions and approximations [6].
